# Intraoperative cartilage analysis of the first carpometacarpal joint - comparison with conventional staging according to Eaton and Littler

**DOI:** 10.1007/s00402-024-05587-w

**Published:** 2024-09-26

**Authors:** Vincent März, Sören Könneker, Martynas Tamulevicius, Peter M. Vogt

**Affiliations:** 1 Department of Plastic, Aesthetic, Hand and Reconstructive Surgery, Hannover Medical School, Carl-Neuberg-Str. 1, D-30625 Hannover, Germany; 2 Department of Plastic Surgery and Hand Surgery, University Hospital Zurich, Zurich, 8091 Switzerland

**Keywords:** First carpometacarpal osteoarthritis, Thumb basal joint, Eburnation, Cartilage, Osteoarthritis, Resection arthroplasty

## Abstract

**Introduction:**

Osteoarthritis of the first carpometacarpal joint is a common pathology of the hand, which may show an increasing prevalence in Germany due to the demographic development. In recent years, not only the current gold standard - the resection arthroplasty of the thumb saddle joint - has been used, but also therapeutic thumb saddle joint arthroscopy. In addition to the patient’s clinical complaints, radiographic diagnostics have been used to decide on treatment, although it has not been proven whether there is a correlation between imaging and clinical complaints.

**Materials and methods:**

Between 2020 and 2022, 20 articular surfaces of the thumb saddle joint undergoing resection arthroplasty for symptomatic basal thumb osteoarthritis were prospectively examined, mapped and compared with preoperative conventional radiographs.

**Results:**

The evaluation of the corresponding articular surfaces showed a higher cartilage destruction at the articular surfaces of the trapezium compared to the first metacarpal. No correlation was found between the stage of osteoarthritis and the Eaton-Littler classification.

**Conclusions:**

Overall, there is a patient-specific heterogeneity of the cartilage damage of the articular surface of the trapezium bone, as well in the metacarpal bone I base in relation to the radiographic diagnosis. Furthermore, an inhomogeneity of the radiographic stage of osteoarthritis of the carpometacarpal joint according to Eaton and Littler in relation to the intraoperatively assessed cartilage damage. The statistical significance of the surgically assessed cartilage damage in relation to the conventional radiographs could not be demonstrated. Thus, the treatment of symptomatic osteoarthritis of the carpometacarpal joint should primarily address the patient’s individual complaints. The radiographic classification according to Eaton and Littler can be used as an additional factor to decide on the surgical procedure but should not delay the therapeutic treatment.

**Level of evidence:**

III.

## Introduction

Osteoarthritis of the carpometacarpal joint I is a common pathology of the hand, which may show an increasing prevalence due to demographic developments worldwide [1]. In recent years, the resection arthroplasty of the thumb saddle joint was the gold standard. Techniques with or without ligament reconstruction were commonly performed. However, surgical interventions for osteoarthritis of the first CMC joint have evolved over time. Therapeutic thumb saddle joint arthroscopy with synovectomy combined with autologous fat grafting has shown promising results in alleviating pain and enhancing function, particularly in the early stages of thumb osteoarthritis [2–4]. Conservative and arthroscopic approaches initially demonstrated good therapeutic outcomes, but they do not prevent the progression of osteoarthritis. Resection arthroplasties, with or without ligament reconstruction or the use of ligamentoplasties, have shown favorable long-term clinical results. However, these procedures are often associated with a reduction in hand strength, leading to a paradigm shift in recent years [5]. Over the past decade, there has been an increasing use and optimization of thumb carpometacarpal joint prostheses [6, 7]. The latest generation of prostheses demonstrates promising survival rates of over 90% [8]. It is anticipated that thumb joint arthroplasty will establish itself as the new gold standard in the future [9]. Until now, in addition to the patient’s clinical complaints, radiographic diagnostics have been used to decide on treatment. However, studies have shown that the intraoperative findings of the cartilage in the thumb saddle joint often show a discrepancy with the preoperative X-ray findings [10]. The aim of the present study was to investigate the extent to which the radiographic staging according to Eaton and Littler can still be used to determine the indications for the treatment of symptomatic osteoarthritis of the thumb saddle joint.

## Materials and methods

**Fig. 1:**
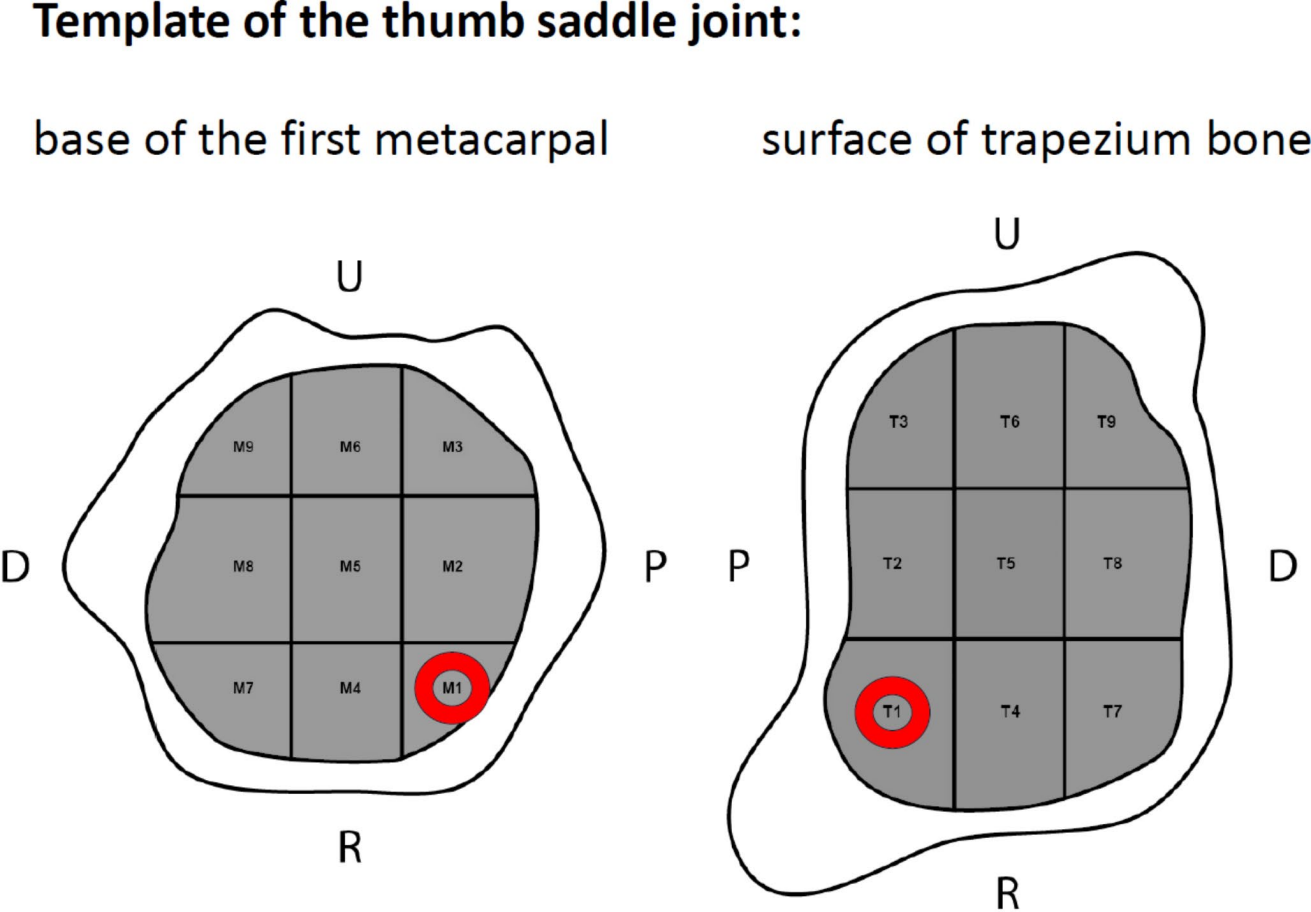
Template for intraoperative assessment of the cartilage status of the thumb saddle joint. The red marking indicates the opposite surface (U = ulnar; P = palmar; R = radial; D = dorsal)

Between 2020 and 2022, 20 articular surfaces of the thumb saddle joint, including the articular surface of the trapezium bone and the base of the first metacarpal bone, were prospectively examined in 20 patients undergoing resection arthroplasty for symptomatic osteoarthritis of the thumb in stages II to IV according to Eaton and Littler, mapped with regard to cartilage damage and compared with preoperative conventional radiographs. Written consent was obtained from the patients for the surgical procedure and the collection of intraoperative data. A positive vote for the study was given by the responsible ethics committee. Approval by the ethics committee was obtained on 25.03.2024, under registration number 11319_BO_K_2024. The operations were performed by different surgeons. The data collection and mapping of the articular surfaces was performed independently of the further intraoperative procedure regarding an isolated trapeziectomy or the necessity of a corresponding arthroplasty with tendon suspension. An anatomical template was designed for intraoperative assessment of the articular surface to describe cartilage of the trapezium bone and the metacarpal base. This template respects the anatomical shapes of the involved bones and divides the articular surfaces into nine corresponding fields of approximately equal size. Two mirrored templates were designed and collected intraoperatively by the surgeons to precisely determine the side to be operated on. All surgeons received preoperative training on the method of use. The nine fields of the template result from three perpendicular paths, oriented from palmar to dorsal and radial to ulnar (Fig. 1).

The cartilage status was assessed for each field. A five-stage classification was used for this, which was based on both the Outerbridge criteria and a possible arthroscopic assessment (Table 1) [11].

**Table 1:** Visual staging of osteoarthritic cartilage damage

Grade	Osteoarthritic cartilage damage
1	none cartilage damage
2	synovitis and soft cartilage
3	partial destruction of the cartilage < 2 mm, cartilage fissures
4	partial destruction of the cartilage > 2 mm, subchondral bone visible

**Fig. 2:**
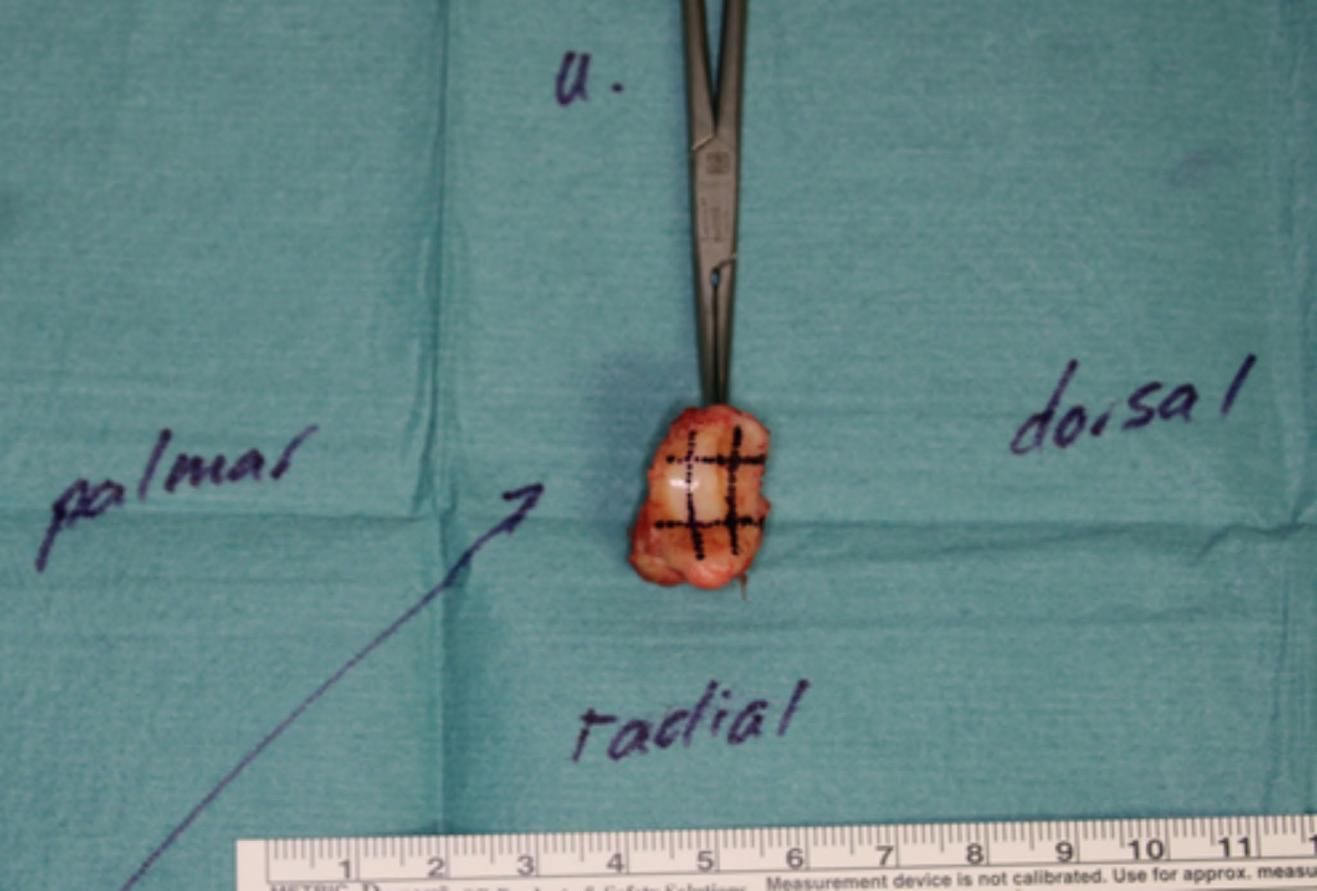
Intraoperative cartilage status of a right trapezium bone resected in toto based on the nine fields. Dorsoradial and radiopalmar with visible subchondral bone (stage 4). Ulnopalmar eburnation (stage 5). Radiographic stage III according to Eaton and Littler

The various fields of the joint components were standardized according to the above-mentioned template from radial-palmar (T1/M1) to ulnar-dorsal (T9/M9) (T1-9 = Os trapezium; M1-9 = MHK I basis). First, the arthrotomy was performed with a resection of the trapezium and the subsequent projection of the cartilage consistency of the trapezium joint surface onto the template according to the stage classification. Figure 2 shows the intraoperative field classification based on an in toto resected trapezium bone.

The joint surface of the first metacarpal bone base was then evaluated by the surgeon. The aim was to resect the trapezium bone in toto, but this could not be guaranteed in all cases. In cases where the carpalia were not resected in toto, the articular surfaces were explored in situ and the cartilage damage was transferred prior to resection. The individual radiographic stages of Eaton and Littler and the cartilage stage of the 20 subjects are shown in Table 2.

**Table 2:** The radiologic stage of osteoarthritis according to Eaton and Littler, stage of cartilage damage per field in the intraoperative template of trapezium bone (T1-T9), and first metacarpal base (M1-M9) in analyzed thumb saddle joints

Nr.	Eaton-Littler classification	T1	T2	T3	T4	T5	T6	T7	T8	T9	M1	M2	M3	M4	M5	M6	M7	M8	M9
1	3	5	5	5	5	5	5	4	4	4	5	5	5	5	5	5	4	4	4
2	3	3	5	5	3	5	5	3	5	5	5	5	5	3	3	3	3	3	3
3	3	4	4	4	4	4	4	4	4	4	1	1	1	3	3	3	5	5	5
4	3	5	5	5	5	5	5	5	5	5	4	4	4	4	5	4	4	4	4
5	2	5	5	5	5	5	5	4	4	4	3	3	3	3	3	3	3	3	3
6	3	5	5	5	5	5	5	5	5	5	2	2	2	2	1	2	2	2	2
7	3	3	3	3	5	5	5	5	5	5	1	1	1	1	1	1	1	1	1
8	3	5	5	5	5	5	4	3	3	3	2	2	2	5	5	5	3	3	3
9	3	4	4	4	3	3	3	3	3	3	4	4	4	3	3	3	1	1	1
10	4	5	5	5	5	5	5	5	5	5	4	4	4	5	5	4	5	5	5
11	3	5	5	5	5	5	5	5	4	4	3	3	3	5	5	3	5	3	3
12	3	1	1	1	1	1	1	2	2	1	1	1	1	1	1	1	2	2	1
13	2	5	3	4	5	3	4	5	3	4	5	2	4	5	2	4	5	2	4
14	3	5	5	5	5	5	5	5	5	5	1	1	1	1	1	1	1	1	1
15	3	5	5	5	5	5	5	5	5	5	1	1	1	2	2	2	3	3	3
16	2	5	5	5	5	5	5	4	4	5	1	1	1	3	3	3	5	5	5
17	3	5	5	5	5	5	5	5	5	5	5	5	5	5	5	5	4	4	4
18	3	5	5	5	5	5	5	5	5	5	5	5	5	5	5	5	5	5	5
19	3	5	5	5	5	5	5	5	5	5	5	5	5	5	5	5	5	5	5
20	2	2	2	2	2	5	2	2	2	2	2	2	2	5	5	2	2	2	2

Statistical analysis was performed using SPSS version 27.0 (IBM^®^ SPSS^®^- for Windows). A value of *P* < 0.05 was considered significant. Spearman’s correlation was used to find the correlation between cartilage damage and X-ray stages of Eaton and Littler. T-test was used to test the correlation of cartilage damage in relation to the respective joint components.

## Results

**Fig. 3:**
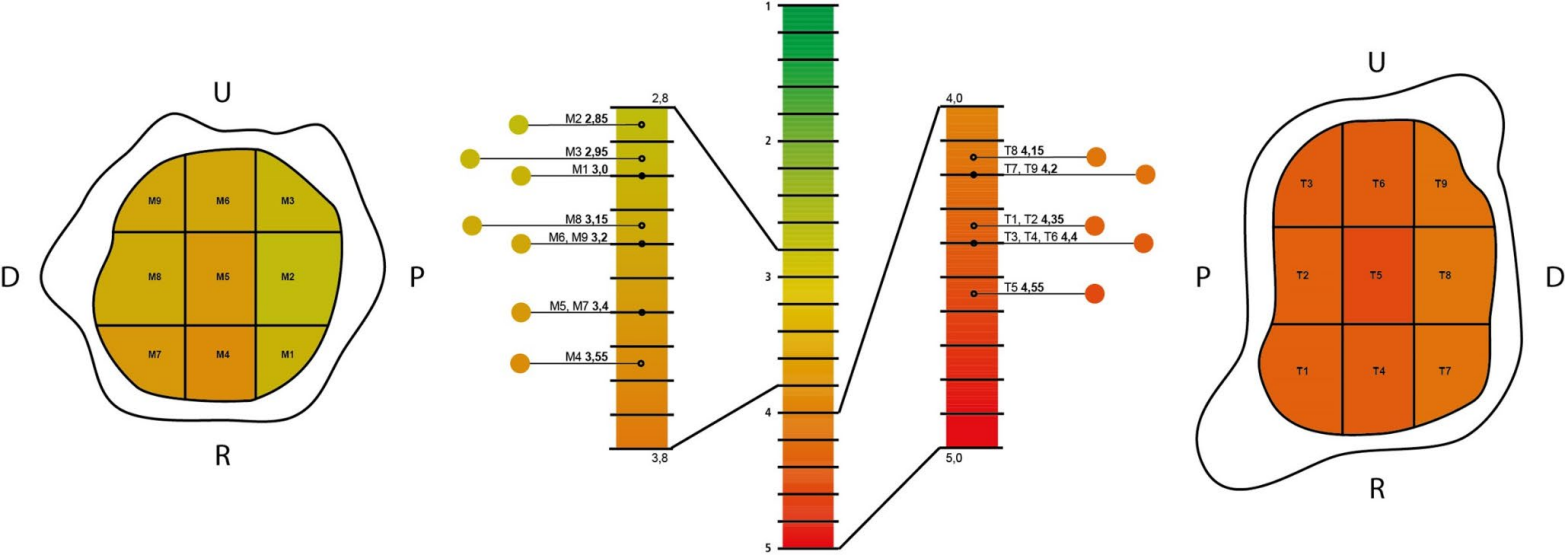
Mean values (color-coded) of the intraoperative cartilage status (stage 1–5) of the 20 thumb saddle joints according to the nine-field classification

A total of 20 thumb saddle joints from 16 women (80%) and four men (20%) were evaluated. The average age of the patients at the time of surgery was 61.8 years (range: 50–80 years). Irrespective of the patients’ handedness, 14 resection arthroplasties were performed on the left (70%) and six on the right (30%) hand. In the preoperative X-ray diagnosis in two planes, four thumb saddle joints were classified as stage II, 15 as stage III, and one as stage IV according to Eaton and Littler. In test subject 12, the least cartilage damage is observed in the region of the trapezium bone, radiographically associated with stage 3 according to Eaton and Littler. Subjects 7 and 14 showed the least cartilage damage in the area of the MHK I base with a radiographic stage of 3. Subjects 18 and 19 showed an eburnation (stage 5) in all fields of the trapezium bone and the first metacarpal base with radiographic stage 3 according to Eaton and Littler. Across all subjects, the articular surface of the trapezium bone is found to be more severely damaged than the base of the first metacarpal in all nine fields (*P* = 0.003) (Fig. 3).

Moreover, in four cases with severe (Grade 4–5) damage on the articular surface of the trapezium bone, only low-grade (Grade 1–3) damage on the first metacarpal was seen. There was no significant correlation between the corresponding fields T1-T9 and fields M1-M9 in the Spearman test. The highest correlation was found in the central palmar joint sections T2/M2 with 0.436 (*P* = 0.055) (Table 3).

**Table 3:** P-values for the comparison of corresponding fields T1-T9 and fields M1-M9. On average, the radiopalmar articular surface (mean value: T1-T3: 4.35–4.4), followed by the radially ulnar middle zone of the trapezium bone, is more destructive (mean value: T4-T6: 4.4–4.6). The dorsal fields show less cartilage damage (mean value: T7-T9: 4.15–4.2)

Zones	T1 vs. M1	T2 vs. M2	T3 vs. M3	T4 vs. M4	T5 vs. M5	T6 vs. M6	T7 vs. M7	T8 vs. M8	T9 vs. M9
Correlation coefficient	0,291	0,436	0,378	0,228	0,388	0,181	0,328	0,282	0,344
P value	0,213	0,055	0,100	0,333	0,091	0,445	0,157	0,228	0,137

In opposition to this, the least cartilage damage is in the palmar area of the MHK I base (mean value: M1-M3: 2.85-3.0), which increases over the central portion (mean value: M4-M6: 3.2–3.55), only to decrease dorsally (M7-M9: 3.15–3.4), without however reaching the damage of the palmar portions (Fig. 3). When evaluating the entire articular surface of the trapezium and the base of the first metacarpal, no statistically significant correlations were found between cartilage damage and stages of the Eaton-Littler classification (for the trapezium *r* = 0.382, *P* = 0.096; for the base of the first metacarpal *r* = 0.173, *P* = 0.465). Moreover, the cartilage damage between the articular surfaces of the trapezium and the first metacarpal did not correlate with each other (*r* = 0.353, *P* = 0.127).

## Discussion

With an incidence of 10%, osteoarthritis of the thumb is one of the most common types in arthritis of the hand. The Rotterdam study by Teunissen et al. found a prevalence of osteoarthritis of the thumb in 7.9% of men and 15.1% of women in the age group 55 to 59. The prevalence of osteoarthritis of the thumb increased to 39.8% in men and 52.5% in women over 80 years of age [12]. In view of demographic developments, this type of osteoarthritis in hand will be an increasing burden for the health systems [6, 13]. Well known and established diagnostic tools and therapeutic methods will have to adapt to this in future. Normally the diagnosis of thumb osteoarthritis is made clinically and radiologically classified into the respective stage according to the Eaton and Littler classification. However, it is unclear to what extent there is a correlation between the clinical and radiographic stages. Therefore, additional research is needed to clarify this relationship. When evaluating the opposing articular surfaces, a clear difference in the qualitative cartilage damage of the trapezium bone surface compared to the base of the first metacarpal could be determined. Pellegrini, on the other hand, described a three times quantitatively greater cartilage damage of the articular surface of the trapezium compared to the metacarpal I [14]. The articular surfaces of the trapezium showed greater cartilage destruction in the radiopalmar column and in the center of the articular surface. Moulton et al. already described a change in the main force acting on the articular surfaces depending on the joint position of the metacarpal joint, whereby increasing thumb flexion shifted the center of the acting force to the center of the trapezium and the palmar articular surface was relieved [15]. In contrast, in our study almost homogeneous cartilage damage in the area of the metacarpal base could be visualized across all nine fields. As early as 2003, Koff et al. divided the joint surfaces into less and more load-bearing areas and visualized the cartilage thickness in 104 donor CMC-1 joints; the lowest cartilage thickness was evaluated here, as in our study, in the radiopalmar region regardless of the stage of osteoarthritis [16]. The cartilage damage found in our study is primarily due to the anatomical construction of the thumb saddle joint with its incongruent articular surfaces and the associated biomechanical incorrect loading of individual articular surface components. In addition to age, gender and secondary diseases, the interindividual damage to the articular surfaces depends on the intraindividual load on the respective thumb saddle joints. This has already been shown by Xu et al. who found a lower joint congruence in female patients compared to male patients and a lower cartilage thickness in female patients [17]. In addition to the incongruent joint surface of the thumb saddle joint, instabilities of the capsular ligament apparatus in particular lead to the progression of osteoarthritis of the thumb and can be clarified in more detail by means of arthroscopy at an early stage if symptoms arise [14]. Furthermore, there is an inhomogeneity of radiographic stage II and III in relation to the cartilage damage detected. Thus, in the initial stage of osteoarthritis, the radiographic joint space may be enlarged due to intra-articular swelling or synovitis, which masks incipient cartilage destruction [18]. It must be assumed that even with minor clinical symptoms and only marginal changes in the radiographic imaging, there is considerably more cartilage damage than would be expected based on the image diagnosis. In 2014, Kalb et al. used a patient example to show that cartilage damage could be diagnosed arthroscopically in the case of symptomatic osteoarthrosis and no correlate in MRI and conventional X-ray diagnostics [2]. Furthermore, Slattery et al. describe that the classification based on the Outerbridge criteria for describing cartilage damage also contains a scattering of findings, which does not allow a reproducible treatment decision to be made [11]. The challenge of accurately assessing cartilage damage in osteoarthritis has long existed. With the improvement of diagnostic radiological techniques, invasive procedures are becoming increasingly unnecessary, particularly in large joints such as the shoulder, knee, and hip [19]. These advancements are expected to continue improving in the future. However, there remains a need to precisely correlate patient symptoms with imaging findings. Despite advancements in imaging technology, moderate cartilage lesions continue to pose difficulties in selecting the appropriate therapeutic approach due to their heterogeneity. In 2013, Löw et al. reported that a more objective assessment of cartilage damage during wrist arthroscopy could be achieved by improving interobserver reliability through intraoperative video recordings of joint conditions, as opposed to individual still images [20]. Regarding the evaluation of cartilage damage in the thumb carpometacarpal joint, Spaans et al. demonstrated in a 2011 study based on 40 radiographs of the thumb carpometacarpal joint that only moderate interobserver reliability could be achieved. Their analysis revealed that radiological stages II-III according to Eaton and Littler showed the lowest interobserver agreement [21]. Similar findings were reported by Kubik and Lubahn, as well as Dela Rosa et al. [22, 23].

The most reliable method for objectively assessing the joint cartilage of the thumb carpometacarpal joint in its entirety is through arthrotomy with complete trapezium excision. Due to the aforementioned divergence in radiological interobserver reliability in evaluating radiographic data, we conducted this study. The poor correlation between Eaton and Littler radiological stages, especially II and III, and more extensive intraoperative cartilage damage suggests that X-rays alone are often inadequate. Therefore, we believe that early surgical intervention is necessary when clinical symptoms persist after at least three months of conservative therapy. Furthermore, the results of this study are intended to support a patient-centered treatment plan. The findings endorse an approach similar to that described by Erne et al., as our results suggest that the suspected cartilage damage may be greater than would be expected based on radiological diagnostics [10]. Therefore, we advocate for stage-specific treatment as follows if the patient’s symptoms persist after conservative therapy: Stage I according to Eaton and Littler requires arthroscopic diagnostics and possibly a synovectomy. Stages II and III according to Eaton and Littler necessitate arthroscopic synovectomy, denervation of the CMC joint, and, if indicated, lipofilling [4, 24]. At present, there is no evidence supporting the intra-articular application of lipofilling, however, it could be a promising option. Especially for differentiating cartilage damage in stages II and III according to Eaton and Littler, an arthroscopic examination should be performed initially, in cases of more advanced cartilage damage, a conversion to an open surgical approach may be required. Stages III and IV according to Eaton and Littler require arthroplasty using the implantation of an total endoprosthesis, or, in the presence of contraindications, resection arthroplasty (RSA). We believe that, whenever possible, the preservation of the thumb carpometacarpal (CMC) joint should be prioritized for as long as feasible. In this regard, advancements in minimally invasive arthroscopy represent a significant progress in the treatment of thumb CMC joint osteoarthritis. Further studies should assess the indications for arthroscopic evaluation of cartilage damage in thumb saddle joint on a case-by-case basis to avoid sometimes unnecessary invasive procedures and provide a more targeted approach for these patients.

As outlined in the aforementioned treatment algorithm, resection arthroplasty (RSA) and total joint arthroplasty should be reserved for the more advanced stages of rhizarthrosis. While current mid-term follow-up results for total joint arthroplasty are promising, they also pose risks such as infections, dislocations, and loosening of the prosthesis [8, 9]. However, it is noteworthy that in the case of implant failure, reverting to the previously established gold standard—RSA—remains a viable option.

## Conclusions

In summary, we found patient-specific heterogeneity of the cartilage damage of the articular surface of the trapezium bone as well as the first metacarpal base. Furthermore, there is a significant inhomogeneity of the radiographic stage of osteoarthritis of the thumb according to Eaton and Littler in relation to the intraoperatively assessed cartilage damage. In our study, no statistically significant correlation could be established in this context.

The treatment of symptomatic osteoarthritis of the thumb should therefore primarily address the patient’s individual symptoms. The focus should be on pain relief with the greatest possible preservation of strength and function of the thumb saddle joint. Our pilot study is intended to emphasize the need for early further diagnostics or initiation of treatment so that patients with osteoarthritic symptoms, but without or with only a slight radiographic correlation, can be treated more quickly and in the appropriate stage. The radiographic classification of osteoarthritis of the thumb according to Eaton and Littler can also contribute to the planning of the surgical procedure but should not delay the early and stage-appropriate initiation of surgical treatment. In our view, the classification according to Eaton and Littler is no longer sufficient for initiating patient-oriented therapy for rhizarthrosis. It remains to be seen how minimally invasive treatment options, such as lipofilling, will perform in the long term compared to the current gold standard, the RSA, and the potentially future gold standard, endoprosthetic joint replacement.
